# Formation of the upper gastrointestinal tract for patients who underwent total esophago-gastrectomy due to caustic ingestion: Case series

**DOI:** 10.1016/j.amsu.2021.102846

**Published:** 2021-09-09

**Authors:** Tran Manh Hung, Thi Phuong Thao Tran, Nguyen Trung Kien, Tran Thi Phuong

**Affiliations:** a Bach Mai Hospital, Hanoi, Viet Nam; b Hanoi University of Public Health, Hanoi, Viet Nam; c Thai Binh University of Medicine and Pharmacy, Viet Nam

**Keywords:** Esophagectomy, Gastrectomy, Formation of the upper digestive tract, Esophageal burns, Stomach burns, Case report

## Abstract

**Introduction and importance:**

No case has been reported regarding esophago-gastrectomy due to caustic chemicals in the literature.

**Case presentation:**

The first case was a 43-year-old woman with BMI 28.5. After one month of taking a weight loss drug called HERBAL, the patient experienced vomiting, and signs of progressive dysphagia, while being unable to eat any solid food. Endoscopy results revealed many scars causing the narrowing of the esophagus, starting from the upper third of the esophagus, 25cm from the dental arch. After 2 months, she lost 26kg (BMI 18.3). Endoscopic reexamination showed the esophagus's stricture, 25cm from the dental arch. X-ray also showed that the esophagus and stomach were completely narrow and atrophied. The second case was a 37-year-old woman who suffered from domestic violence and drunk about 50 ml of toilet detergents to commit suicide. After one month, the patient went through dysphagia and was unable to eat. Esophageal endoscopy showed that the esophagus was narrowed in the upper third part, 20cm from the dental arch, which led to the inaccessibility of the conventional insertion tube that required nasoscope instead.

**Clinical discussion:**

The results demonstrated many ulcer scars, retraction inside the esophagus and stomach, abnormally small volume of stomach, narrowing cardia, and pyloric stenosis. In both cases, thoracoscopic surgery was performed for esophago-gastrectomy, and the upper gastrointestinal tract was subsequently reconstructed using the ileum-right colon.

**Conclusions:**

The ileum-right colon segment is a part that can be used to reconstruct the upper gastrointestinal tract following esophago-gastrectomy.

## Highlights

• Esophagus and stomach were damaged due to caustic ingestion, causing the stricture, narrowing and tightening of both organs.
• In these cases, the upper gastrointestinal tract was reconstructed via esophagus-ileum anastomosis in the left-hand neck.
• Ileocolic parts can be used to reshape the digestive track following total esophago-gastrectomy.

## 1 Introduction

Erosive lesions of the gastrointestinal tract are commonly seen in developing countries as a result of accidental ingestion of acids/alkalis or intentional suicides. Caustic chemicals can damage any segment of the gastrointestinal tract, but most commonly affected parts are the upper gastrointestinal tract, including the pharynx, larynx, esophagus and stomach. Concurrent damage of both esophagus and stomach accounted for 20%–62.5% of all cases [[1], [2], [3], [4], [5]]. To the best of our knowledge, there have been very few reports of esophagectomy for caustic substance-related cases and ones of esophageal reconstruction with colon [6,7]; however, once these chemicals have severely damaged both stomach and esophagus, the resection and reconstruction of the upper gastrointestinal tract is imperative. This is an extremely rare case and no report in the literature has ever been claimed in terms of esophago-gastrectomy due to caustic chemical-associated damage. Here, we reported the first two cases of women with simultaneous burns of both esophagus and stomach due to caustic chemicals, which subsequently caused retraction, stricture, and tightening of the entire esophagus and stomach, before their surgeries of esophago-gastrectomy and the reconstruction of their upper gastrointestinal tracts were successfully operated at Bach Mai Hospital. These case reports have been conducted in line with the SCARE Criteria [8].

## 2 Presentation of case

### 2.1 The first case

The first clinical case was a 43-year-old female patient, working in the business field. Her height and weight at that time were 1m60 and 73 kg (BMI 28.5), respectively. Previously, the patient was completely healthy, and did not consume any tobacco or alcohol. She had taken a weight loss drug called HERBAL 450mg at a dose of 2 tablets/day without using any other medicines. After one month of taking the drug, the patient experienced vomiting and signs of progressive dysphagia, while she was only able to take porridge and milk, and was unable to eat any solid food before going to Internal Department of Bach Mai hospital for examination.

**Fig. 1:**
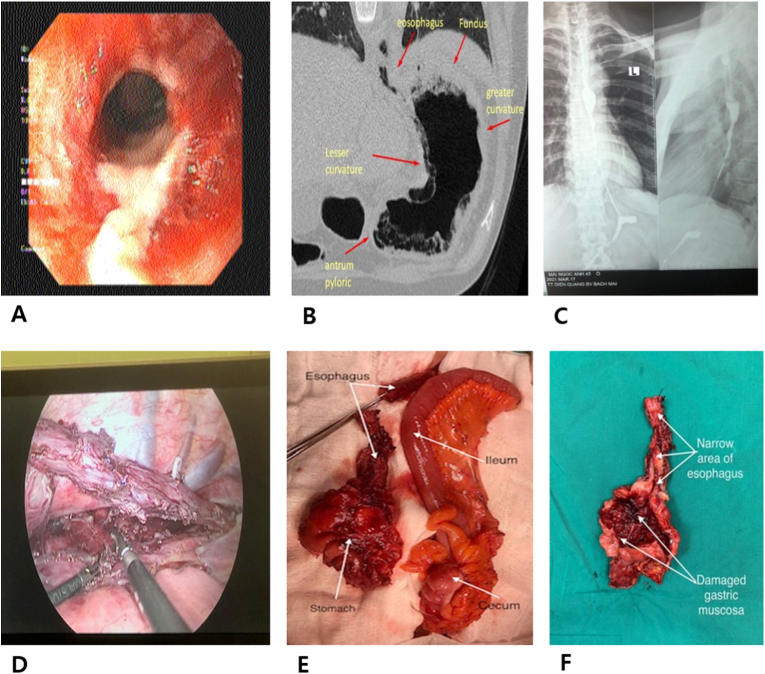
Damages to esophagus, stomach and surgical methods of the first case. A, B. Damage to esophagus and stomach one month after drug consumption; C. Preoperative image of esophagus and stomach; D. Thoracoscopic esophagectomy; E. The resection of stomach and esophagus (left), the segment of ileum and right colon for upper digestive tract reconstruction (right); F. Damage to the esophagus and stomach (atrophy and narrowing) caused by weight loss drugs.

Endoscopy results revealed many scars causing the narrowing of the esophagus, starting from the upper third of the esophagus, 25cm from the dental arch (Fig. 1A). A thoracic and abdominal computed tomography (CT) showed wall thickening with gases between layers, and especially a marked volume loss of the stomach (Fig. 1B). The patient also undertook an endoscopy and esophageal dilation, but no promising results were shown, then being transferred to Department of Surgery after 2 months from the time of taking weight loss pills that she lost 26kg (BMI 18.3). She was provided with total parenteral nutrition (an energy intake of 2,500kcal/24 hours) and fluid and electrolyte replenishment. After two weeks, the patient gained 4 kg, while her biochemical and hematological tests revealed were within normal limits. Endoscopic reexamination showed that the esophagus was stricture, 25cm from the dental arch. X-ray also depicted that the esophagus and stomach were completely narrow and atrophied, with very little Barium sulfate reaching out the stomach (Fig. 1C). Therefore, the patient was diagnosed preoperatively with burnt scars caused by caustic chemicals, leading to the narrowing of the entire esophagus and stomach. Therefore, esophago-gastrectomy was indicated to reconstruct the upper gastrointestinal tract using the ileum-right colon. The operation was performed on April 6th, 2021, 10th day of hospitalization.

The patient was placed in the left lateral-prone position, 45 degrees and underwent the right thoracoscopic approach with 4 trocars. The thoracic esophagus was thick and adherent, so we decided to dissect it to, free the entire part (Fig. 1D), insert a right-side chest tube, and close the trocar holes. The patient was moved to the supine position for laparotomy with an incision along the linea alba, above and below the umbilicus, being made. Stomach was found to be decreased to a very abnormal size (10 × 5cm), and its wall was inflammatory, thickened and adherent. Opening of the stomach depicted that the gastric mucosa had many retracting ulcer scars, which could not be persevered, but required the removal of the entire stomach. The J-line incision in the left-hand neck was done so that the cervical esophagus was mobilized. We then cut across the thoracic esophagus above the narrow point, then removed the part below esophagus and stomach and brought it out to the abdomen. After the mobilization of the ileum and right colon segment, the right colic artery, middle colic artery, and marginal artery of the (ileocolic) colon were preserved by being ligated, at its origin. We did the transverse section's cut through the ileum, 8 cm far from the Bauhin valve. The intestinal segment, consisting of the short ileum and right colon part, was pulled through the posterior mediastinum to the neck, and then it was anastomosed to the cervical esophagus side-by-side using a 75 mm stapler, while, the right colic flexure (the right transverse colon) was joined with the proximal jejunal loop by an end-to-side anastomosis using suture safil 4/0. The remained jejunal part was anastomosed to the long transverse colon side-by-side using a 75mm stapler. (Fig. 1E and F).

The surgery went smoothly, taking place in 5 hours, and was performed by a team of 4 surgeons with the primary surgeon having 26 years of experience in the surgical specialty. 30 minutes after of surgery, the patient was awake and well responded. On the 5th day post-surgery, she experienced symptoms of pneumonia and the culture of bronchial fluid was positive with the appearance of Pseudomonas aeruginosa bacteria. She then was treated with Meronem 3 g/24 hours in combination with the vibration therapy, breathing exercises, reposition, and enteral feeding with 2,500 kcal/24 hours by nasogastric tube. On the 12th day post-surgery, the patient's condition was stable, the nasogastric tube was removed, and full oral feeding was administered. The patient was discharged from the hospital on April 24th, 2021 (18 days after surgery).

### 2.2 The second case

The 37-year-old female patient was a farmer, 1m54 tall and weighing 63kg (BMI 26.5). The patient suffered from domestic violence and drunk about 50 ml of toilet detergents with the main component of Sodium hypochlorite to commit suicide. After 8 hours of drinking, she was transferred to an Emergency Department at the Poison Control Center of Bach Mai hospital. Herein, the patient was placed a gastric tube to treat esophageal stricture, together with nutrition feeding, while being monitored regarding acute complications of burns.

**Fig. 2:**
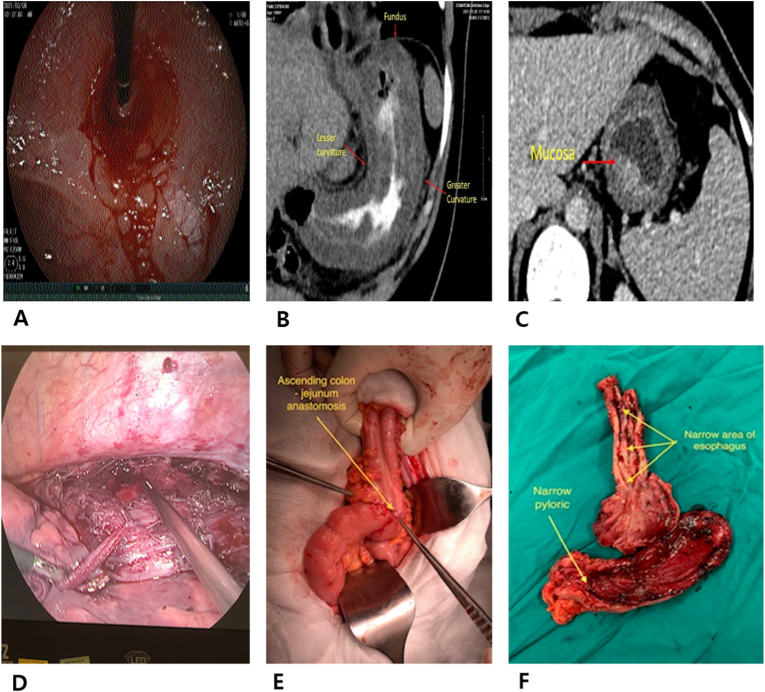
Damage to esophagus, stomach and surgical methods of the second case. A, B, C. Damage to the esophagus and stomach one month after drinking toilet cleaners; D. Endoscopic surgery of thoracic esophagectomy; E: Anastomosis of transverse colon-jejunum; F. The atrophic and narrowing parts of the stomach and esophagus was removed, being caused by toilet cleaning agents.

**Fig. 3:**
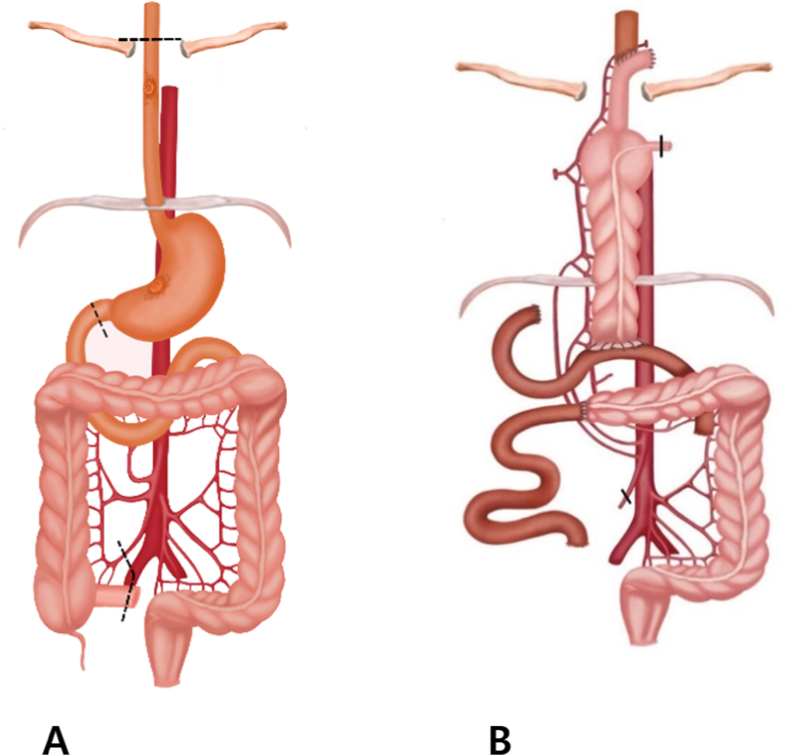
Surgical procedure illustration that describes of the upper gastrointestinal tract reconstruction using the ileum-right colon segment for patients who were required to total esophago-gastrectomy.

After one month of receiving treatment there, the patient experienced dysphagia and was unable to eat. Esophageal endoscopy showed that the esophagus was narrowed in the upper third part, 20 cm from the dental arch, which led to the inaccessibility of the conventional insertion tube that required nasoscope instead. The results demonstrated many ulcer scars, retraction inside the esophagus and stomach, abnormally small volume of stomach, narrowing cardia, and pyloric stenosis (Fig. 2A). Abdominal CT scan showed that there were thickened gastric wall with gases between layers and a smaller stomach volume (Fig. 2B & C). The patient was then transferred to the Department of Surgery for an inability to eat and drink, weighing 47kg (BMI 19,8). Herein, the patient underwent laparoscopic surgery, including gastrojejunostomy and gastrostomy for tube feeding. After one month of tube feeding, she gained 4kg. The endoscopic reexamination showed an extremely esophageal stricture, 20 cm from the dental arch, making it impossible for the tube to be placed, even by using the nasoscope. The patient was indicated for surgery with a preoperative diagnosis of scarring strictures plus entire retraction of esophagus and stomach, which were all caused by caustic ingestion. The surgery occurred on May 4th, 2021 with steps performed similar to those of the first case (Fig. 2D & F). The only difference was that anastomosis of the thoracic esophagus and ileum segments was taken in an end-to-end approach using suture safil 4/0, and the anastomosis of the transverse colon with the proximal loop of the jejunum was also carried out in the same way. (Fig. 2E). Surgical procedure illustration of both cases was shown in Fig. 3.

The surgery was performed by the same team as in the first case, lasting 5.3 hours, without any complications. In the first 3 days post-surgery, the patient was supported by total parenteral nutrition with promising responses. On the 4th day post-surgery, she had bowel movements again and underwent enteral feeding with 2,500 kcal/24 hours though nasogastric tube. From what was shown in the Chest X-ray result (PA), the costophrenic angles were acute and sharp, both lungs were well-expanded, and the chest tube on the right was then removed. On the 7th postoperative day, the nasogastric tube was removed, the patient was indicated for the enteral nutrition with liquid diet and had good responses. During the treatment, she was mentally comfortable and fully complied with medicinal therapies without any psychological support. The patient was discharged on 9-day post-surgery.

**Fig. 4:**
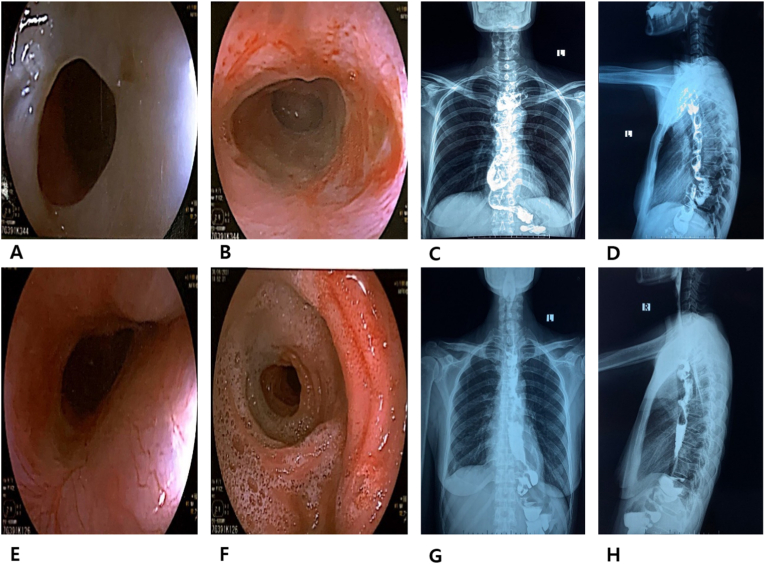
Endoscopic and X-ray images at follow-up examination, 2 months after the surgery. First case: A esophago-ileal anastomosis; B jejunum-colon anastomosis; C, D. Chest X-ray of the esophagus-ileum-colon-jejunum segment with Barium Sulfate (PA and lateral views); Second case: E. esophago-ileal anastomosis; F. Valve Bauhin; G, H. Chest X-ray of the esophagus-ileum-colon-jejunum segment with Barium Sulfate (PA and lateral views).

Both cases were scheduled for follow-up examination, with endoscopic examinations conducted after 2 weeks, 1 month, and 2 months of being discharged. The results of endoscopy and the scanning of the upper gastrointestinal tract at two months after surgery in both cases showed that both the ileocolic and jejunocolic anastomoses were in good condition with food normally passing through. The barium sulfate was administered and gradually reached the esophagus, ileum, colon, and jejunum (Fig. 4). Both patients were able to eat and drink without any signs of reflux, having gained 2–3 kg. Their families were satisfied with these results as their beloved ones were healthy and could eat again after many months of being weak and unable to eat food.

## 3 Discussion

According to Gerald F. O’ Maylley, around the world, 80% of caustic chemical poisonings occur in young children, which are usually accidents due to negligible consumption of these substances and are benign most of the time. In adult, caustic chemical poisoning is often a result of committing suicides, with large amounts of substances consumed that can be life-threatening. Sources of caustic chemicals include solids, liquids and toilet detergents [9]. The damaging sequence progresses through 5 stages: necrosis arising from the acute phase in the first few days, small vessel thrombosis, mucosal erosion, followed by bacterial invasion and the destruction of fibroblasts within the first week; after which collagen deposition appears at week 2, scar formation begins at week 3 and this eventually leads to the stricture and the tightening of the gastrointestinal tract over the next several weeks, even when initial symptoms are mild and adequately treated [1,4]. In the two cases shown in our report, the second case was a suicide attempt by drinking toilet detergents, which is a commonly cited cause in the medicinal literature. However, in the first case, the upper gastrointestinal burn was caused by weight loss pills (named HERBAL), advertised as a derivative of natural herbs and sold widely online with no clear evidence of its origin and its chemical compositions. This is an emerging and alarming issue in our country. We have never encountered the situation before and there is no such case reported in the literature yet. The two cases in our report both had concurrent esophagus and stomach burns. Although both patients had no acute complications, the impacts were extremely severe and complicated, including the stricture, retraction of both the esophagus and stomach. These described lesions are rare and have not been mentioned in any reports of caustic ingestion-related gastrointestinal damage, which definitely required the total esophago-gastrectomy.

The question raised is which part of the gastrointestinal part should be used to replace ones taken out as a result of esophago-gastrectomy to ensure that the tract is still long enough with good blood vessels maintained and reflux limited. Using the stomach is a potential solution, predominantly applied for patients who have to undergo esophagectomy as the stomach is of good length, a good vascular supply, and requires only a single anastomosis. However, when the stomach is not in presence (due to being absent or being removed along with the esophagus), the surgeon must choose which part of the digestive tract can be employed to replace both the removed stomach and esophagus. There have been studies reporting this issue, such as one opting for jejunum for replacement following the removal of both esophagus and stomach by Maier A et al. [10]. Honda et al. [11] reported an endoscopy surgical case of both thoracic esophagus and stomach removed and the right colon was used to reconstruct the upper digestive tract by connecting the esophagus to the colon at the thorax for the treatment of lower third esophageal cancer and middle third of stomach cancer. Bassiouny IE et al. [6] utilized the colon to replace esophagus that was already burnt out due to caustic chemicals in children, and with an aim to reduce any chance of reflux, the author also suggested connecting the colon to the front part of the stomach. Bita Shahbazzadegan et al. reported two cases of caustic ingestion-related gastric and esophageal burns that underwent surgical replacement of the esophagus with the left colon and transverse colon with anastomoses of esophagus-transverse colon at neck and left colon-stomach in the abdomen. Nevertheless, stomachs in both cases were still in preserved conditions [7].

Unlike what was implemented for the two cases reported by Bita Shahbazzadegan, the two cases in our report showed signs of both the esophagus and stomach seriously damaged without any preserving chances, so we came up with resecting those. We decided to use the ileum-right colon segment for the upper gastrointestinal reconstructive surgery following the resection of both esophagus and stomach burns due to caustic ingestion. To our best knowledge, these are the first two cases that both esophagus and stomach were resected concurrently due to the consumption of corrosive chemicals, which were then reformulated using ileum-right colon segment in the literature. The colon has been commonly claimed as a potential part to replace the esophagus and stomach in a wide range of previous medicinal reports [6,7,11,12]. In our surgery, we used an additional 8 cm of ileum connected with Bauhin valve to eliminate any refluxing symptoms, whereas both the cardia and the pyloris were removed to increase the quality of life for patients post-operatively. In fact, both patients included did not showed any refluxing problems under examination. We also had experience of performing esophago-gastrectomy, which was then reconstructed with the ileum-right colon segment in a patient with concurrent esophageal squamous cell carcinoma and gastric adenocarcinoma in May 2020 [12]. In that case, the patient was examined periodically, and 15 months after the surgery, she was totally healthy with normal eating patterns and no reflux. Furthermore, examination results showed that anastomosis of esophagus-ileum was smooth, not narrow; Bauhin valve and the colon were in normal conditions, along with good circulation of drugs along the path of esophagus-ileum-colon-jejunum. Compared to that case, in this case series reported, we did not follow the Orringer technique to dissect the thoracic esophagus; instead, we performed thoracoscopic surgery with right-side approach to dissect thoracic esophagus. This has created an important advantage that even though the esophagus tissue was thickened, adherent in response to inflammation and fibrosis, we still had good control without any intraoperative complications, or there were not any unfortunate events involved. This was a minimally invasive surgery, so it helps enhance the recovery of postoperative patients.

Based on these outcomes, we have found out that using the ileum and right colon segment to replace the resected upper gastrointestinal parts is safe and effective. It may be one of the most potential options for the upper gastrointestinal reconstruction following the esophago-gastrectomy due to caustic ingestion for these reasons: 1) The ileum and right colon segment is an intestinal tract with the optimal length to connect the thoracic esophagus (or even from the oropharynx level) to the intestinal part inside the abdomen. 2) The ileocolic segment has a rich blood supply that ensures the nourishment of anastomoses and the function of food transportation. 3) The Bauhin valve preservation is significantly helpful to limit risks of reflux.

## 4 Conclusion

Caustic ingestion can cause serious damages to both the esophagus and the stomach, which are later required to be simultaneously resected. The ileum-right colon segment is a part that can be used to reconstruct the upper gastrointestinal tract following esophago-gastrectomy.

## Provenance and peer review

Not commissioned, externally peer-reviewed.

## Availability of data and material

Not applicable.

## Ethics approval

Writing and publishing this case report was approved by Bach Mai hospital.

## Sources of funding

The authors did not receive support from any organization for the submitted work.

## Authors’ contribution

Conceptualization: TMH; Data curation: TMH and NTK; Formal analysis: TMH; Investigation: TMH; Methodology: TMH; Project administration: TMH; Resources: TMH; Software: TMH; Supervision: TMH and TTP; Validation: TMH and TPTT; Visualization: TMH, TPTT, and NTK; Roles/Writing - original draft: TMH and TPTT; Writing - review & editing: TMH, TPTT, NTK, and TTP.

## Consent for publication

Written informed consent was obtained from the patient for publication of this case report and accompanying images. A copy of the written consent is available for review by the Editor-in-Chief of this journal on request.

## Registration of Research Studies

Name of the registry:

Unique Identifying number or registration ID:

Hyperlink to your specific registration (must be publicly accessible and will be checked):

Our case report is not First in Man study.

## Guarantor

Tran Manh Hung.

## Declaration of competing interest

The authors have no relevant financial or non-financial interests to disclose.
